# Patent data access control and protection using blockchain technology

**DOI:** 10.1038/s41598-022-05215-w

**Published:** 2022-02-17

**Authors:** Hui Li, Ming Li

**Affiliations:** National Intellectual Property Administration, Beijing, 100088 China

**Keywords:** Information technology, Computer science

## Abstract

The purposes are to develop the patent data profoundly, control the data access process effectively, and protect the patent information and content. The traditional patent review systems are analyzed. For the present patent data security and privacy protection technologies and algorithms, the patent information data are stored on different block nodes after data fragmentation using blockchain technology. Then the data are shared using the data encryption algorism. In this way, data access control can be restricted to particular users. Finally, a patent data protection scheme based on privacy protection is proposed. The security of the scheme and the model performance are verified through simulation experiments. The time required to encrypt 10 MB files with 64-bit and 128-bit data is 35 ms and 105 ms, respectively. The proposed re-encryption algorithm only needs 1 s to decrypt 64 KB data, and only 1% of the data needs asymmetric encryption. This greatly reduces the computational overhead of encryption. Results demonstrate that the system can effectively control the access methods of users, efficiently protect the personal privacy and patent content of patent applicants, and reduce the patent office cloud computing overhead using the local resources of branches. The distributed storage methods can reduce the cloud system interaction of the patent office, thereby greatly improving the speed of encryption and ensuring data security. Compared with the state of the art methods, the proposed patent data access and protection system based on blockchain technology have greater advantages in data security and model performance. The research results can provide a research foundation and practical value for the protection and review systems of patent data.

## Introduction

At present, the world has been changing tremendously. China is accelerating the construction of a new development pattern in which its big domestic cycle is the subject, and the domestic and international double cycles promote each other. As the core support of the digital society, the cloud platform will become a significant focus of opening up the “double cycles”^1^. As an important link, the cloud platforms have developed rapidly in various fields such as the internet, government affairs, finance, transportation, logistics, and education, accelerating the implementation of “all cloud services all services cloudification” and fully empowering urban governance modernization and high-quality economic development. Big data has penetrated every industry and business functional area and has become an influential production factor^2^. Cloud computing and big data have made the entire society increasingly dependent on science. In the era of big data, “cloud government affairs” depending on big data has become a new model of social governance. Big data has become a key target of network attacks due to its huge value and centralized storage management mode. Blackmail attacks and data leakage problems in big data are becoming increasingly serious^3^. Data concentration leads to a greater impact on internal leakage risks, data distribution leads to a greater impact on external leakage risks, and an increase in data-associated personnel will also cause greater risk problems^4^. He et al. pointed out that with the increasing demand for data sharing in the era of big data, user data cannot be better protected, and user privacy is at risk of leakage because the data shared by users on the internet depend on third-party platforms^5^. As of now, many studies about data privacy protection have been reported; however, these research results are difficult to apply into subdivided fields^6^. Therefore, the access control and protection of data have become scientific problems that need to be solved urgently.

Patent data contain user privacy. The current policy results in that each patent submission and review involves private information, such as the certificate number, telephone number, and detailed address of the applicants. Second, the patent data also contain key technological innovation information before its disclosure. Thus, leakage will cause significant loss of interest to the applicants; in the meantime, patent review conclusions and invalid information on reexamination also involve significant interests of relevant parties^7^. Joung and Kim pointed out that the current internal protection of patent data lay in the development of a new generation of patent review and retrieval systems, which greatly improved the security of patent data access by adding cloud computing and big data processing methods^8^. Bhattarai et al. suggested that the small number of national intellectual property bureaus and effective processing capacity often led to longer processing and review of patent data. Therefore, while adopting traditional data access security, not only should protecting the security and privacy of data storage focused but the distributed deployment should also be supported while ensuring performance and security. In this way, the efficiency of patent review could be improved^9^. In particular, the continued development of the review centers will place higher requirements on the data processing functions and access speed of the patent review systems with examiners all over the country^10^. Such a trend requires a new patent review system that can quickly distribute processing and protect data privacy.

Therefore, a scheme strategy based on data fragmentation and encryption is proposed based on studying the structure of the traditional patent review systems. This scheme uses blockchain technology to store the patent information data in different block nodes after data fragmentation. The innovation is that the encryption algorithm is used to process the privacy of patent data and content, and the hierarchical storage of data is realized. On this basis, safe and effective patent data is constructed to protect the scheme. This provides a research basis and practical value for the protection and review of patent data.

There are five sections in total. The first section is the “Introduction”, in which the importance of data access control and protection and the problems in patent data protection are put forward, and the research ideas are determined. The second section provides a “Literature review”, in which the research framework is clarified according to the development of patent information, classification and characteristics of patent data, the requirements for patent data security in the new stage, and the current research progress. The third section introduces the Research methodology, in which the data fragmentation and encryption algorithm involved are explained, the corresponding storage and encryption model is proposed, and the data access control and protection model is constructed based on patent technology. The fourth section is the “Results and discussions”, in which the proposed model is analyzed using examples. The model’s safety performance and the algorithm’s advantages are summarized, and the proposed scheme is compared with the method proposed in the previous research. The fifth section is the “Conclusion”, in which the key results, actual contributions, limitations, and prospects are illustrated.

## Literature review

### Development of patent information

Cloud computing can provide global users with computing power and storage services via the internet and present the hardware foundation for internet information processing. Big data uses increasingly mature cloud computing technology to obtain valuable information from the vast internet data for information induction, retrieval, and integration, providing a software foundation for internet information processing^11^. Cloud computing is the virtualization of hardware resources, while big data is the efficient processing of massive amounts of data. In China, the public’s awareness of intellectual property has continued to increase with the in-depth advancement of the intellectual property strategy in recent years. As a result, the number of patent applications has increased rapidly. Such a situation has also posed new challenges to the patent reviewing the ability of the China National Intellectual Property Administration^12^. Intellectual property protection is the “rigid need” for innovation-driven development. Intelligence is the most important technical pillar to strengthen the business support capabilities of information systems. The intelligent upgrade project of the national patent review and retrieval system adopts a micro-service framework of “high cohesion and low coupling” for the resource nation of infrastructure and the application of service in the way of “platform + module”^13^. Apply artificial intelligence servers to basic cloud platforms, big data platforms, business modules, business applications, security systems, and overall operation and maintenance can promote the improvement of patent retrieval quality and efficiency. Consequently, the upgrade and optimization of system functions and technical capabilities can help the sound development of patent review services.

### Classification and characteristics of patent data

Patent data are classified into four categories: public data, non-public data, structured data, and unstructured data. Patent data include key equipment parameter data, which are in line with national technology and industry standards, with data processing content and purposes. Patent data are characterized by many small files in unstructured data. There are more than four million applications every year, and 160 million documents are generated, including XML (EXtensible Markup Language), PDF (Portable Document Format), and other formats. The same data are rarely accessed by multiple users at the same time, with no hot data on the internet, and the cache effect is not apparent. The same user will repeatedly access and may modify the data and files of the same patent within a period. There may be 2–3 departments accessing data and files simultaneously and requesting that the modified information can be acquired by other parties promptly. Data are accessed for a long period/have a huge amount. Statistics can show the amount of various data. Data are concentrated in the patent office, and all branches need to access the data via a large centralized system. Data leakage will significantly harm the national economy^14^.

### Data security and privacy protection technology

At the current stage, a new generation of patent review and retrieval systems combining the advantages of cloud computing and big data needs to be established because of the new challenges in security and privacy. Especially after the high-value and high-privacy data are concentrated, the security risks become greater, and further steps are needed to prevent the risk of data leakage by system operation maintenance providers and data platform vendors^15^. The new generation of the system can support the security of future data distributed deployment in the various examination and consultation centers and agencies, fully utilize the computing power of various external agencies, and reduce the pressure on patent office calculations and networks. In recent years, major regions and developed countries in the world have successively formulated and promulgated privacy protection strategies, schemes, and policies, the approaches of protecting data security and personal privacy under the big data background, and the development plans for big data construction. Also, they have strengthened the utilization and security protection of big data to occupy the commanding heights of the international competitiveness of big data technology. The context-sensitive access method can improve the security status, reduce the complexity of user operations, and enable users to log in with any device at any place^16^. It introduces a security key developed by Google to verify its integrity and is designed to protect users from the potentially damaging effects of credential theft. Cloud Armor DDoS and application defense services provide access control functions based on geographic location. This new feature allows enterprises to control the access to their services based on the geographic location of the client. It can also set a whitelist to block malicious traffic, deploy preset prevention rules for injection and cross-site scripting attacks, and implement flow control based on user-selected parameters in the third to seventh layers^17^. However, the above techniques are not suitable for big data security and privacy protection requirements in the patent field.

## Patent data access control using blockchain

### Overall framework

**Figure 1:**
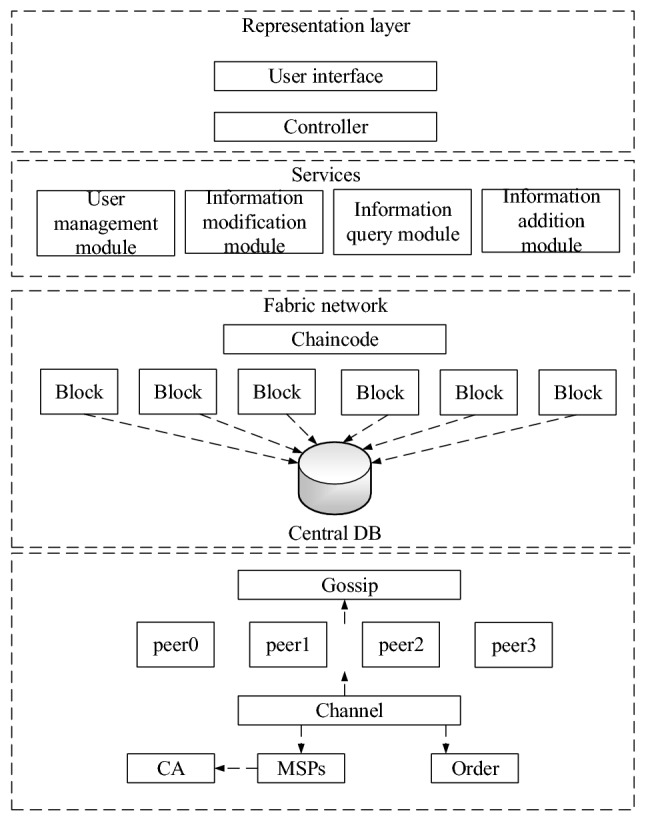
Patent data access control and protection framework based on blockchain technology.

A research framework is proposed based on the above analyses. User privacy and undisclosed patent data are stored using data encryption, data fragmentation, and other means to achieve secure storage. Due to the fragmented storage of documents, the data stolen from the storage device cannot be used. For the branch data fragmentation storage, 99% of the branch are stored on the ground, and 1% is securely stored in the patent office for anti-espionage and anti-electronic monitoring purposes. The framework can prevent system operators and data managers from obtaining data in violation of regulations, prevent cloud service providers from embezzling and tampering with user program codes, private data storage, and backup data in cloud hosts, cloud storage, and other facilities, and prevent data leakage from peripheral organizations. It can ensure data security, improve operational efficiency, and provide security support for the localized storage of data in the data centers of the auditing associations across the country by combining the existing patent data through fragmentation and encryption technology. This framework is written as DS-EA (Data Segmentation Encryption Algorithm) model. The detailed system architecture is shown in Fig. 1.

The entire system adopts a three-layer design. From top to bottom comes the user interface layer, the controller layer, and the Hyperledger Fabric blockchain network data layer. Users only need to focus on the software itself and simply and directly process and operate the data according to their needs. The MVC (Model View Control) design with the front and back ends separated has the characteristics of low cohesion and high coupling, which is easy for system development and maintenance, with higher scalability. The interaction layer is responsible for business data display, data visualization, process visualization, and data initiation operations. It is designed and created according to the data model and business model. The user interface layer is displayed in front of the users and can visually interact with the users. The interaction layer converts the users’ operations of the system into information and transfers it to the controller layer. The controller layer contains the basic modules of personnel information management to implement the core business logic of the system. It is divided into four functional modules: user management module, information query module, information modification module, and information addition module. The chain code is used for smart contract operations on blockchain data.

### Data fragmentation scheme

**Figure 2:**
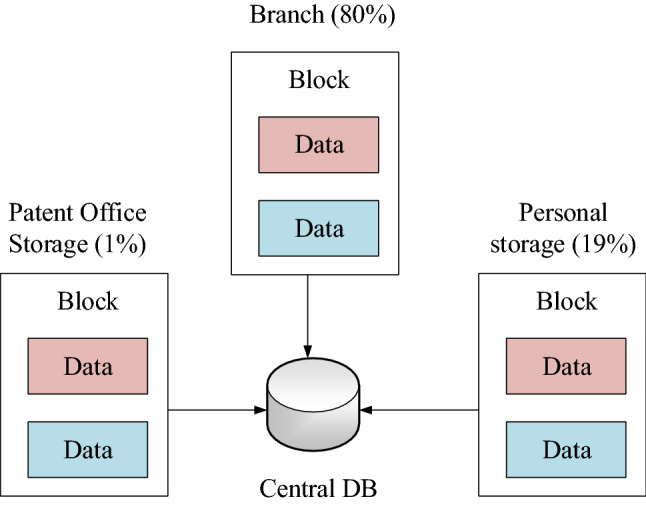
Block data fragmentation storage.

Data in most business scenarios can be divided into core data and non-core data according to the degree of confidentiality. For example, in personnel information management, ID card, phone number, and home address are core data, while age, gender, and department are non-core data. As shown in Fig. 2, each type of data is fragmented into two copies: the patent office storage (1%) and the branch storage (99%). Part of the core data is stored in the blockchain, and the remaining large amount of non-core data can be stored in the central database outside the blockchain after fragmentation. Since the storage of data in the central database is extremely scalable compared to the blockchain, the storage performance is much higher than that of the blockchain. Therefore, the block is greatly reduced. The chain storage pressure reduces the data redundancy of the blockchain and makes the storage resources more efficiently used^18^.

**Figure 3:**
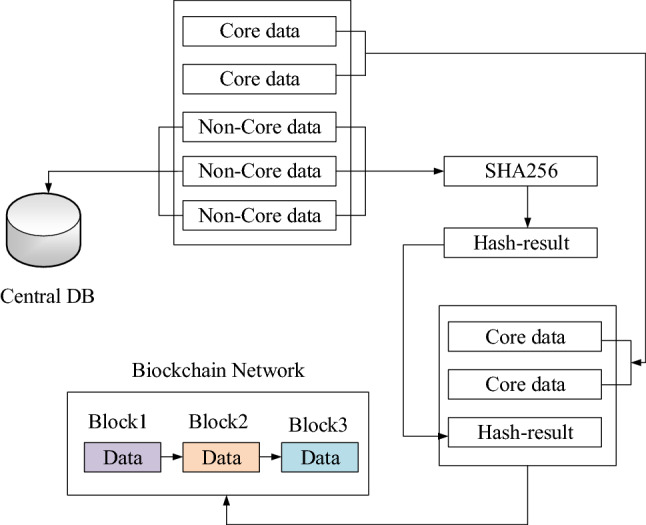
The flowchart of fragmentation storage.

Figure 3 displays the process of storing data fragmentation in the blockchain and the central database. These data are packaged into a data block and stored in the blockchain network. Other non-core data are first subjected to SHA256 calculation. Then the obtained hash value is also packaged and stored together with the core data block into the blockchain network, used as a certificate for data integrity verification in subsequent queries. Subsequently, the non-core data are stored in the central database. The core data stored in the blockchain can utilize the characteristics of the blockchain to achieve tamper-proof and traceable effects. The non-core data stored in the central database can effectively reduce the redundancy of the data on the blockchain.

### Data encryption scheme

**Figure 4:**
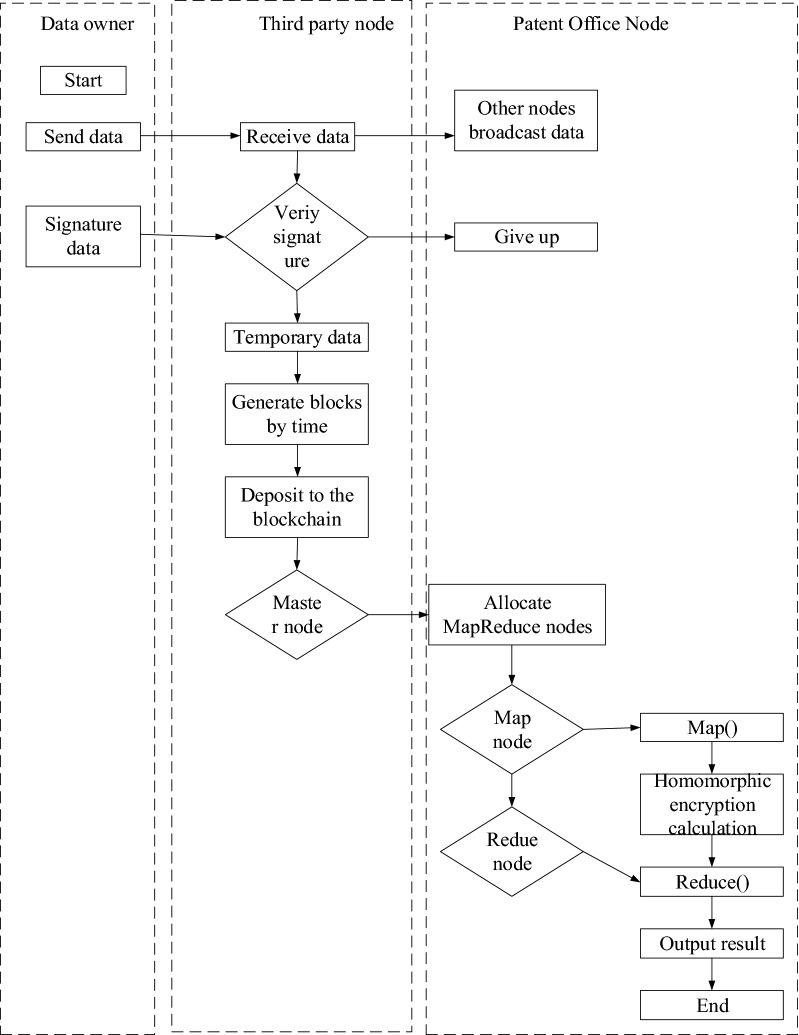
The flowchart of system encryption.

The role-based access control technology: models based on permissions, users, roles, and data classification cannot avoid the data administrator risks and data file embezzlement risks. Big data access control based on cryptography: cryptography can provide higher security, while encryption brings a computational burden and affects performance. The detailed data encryption process is as follows:

1. Initialization of data encryption: the data owner divides the data resource *F* that needs encryption protection into data blocks according to different access control requirements. Then it uses the symmetric encryption key for plaintext encryption *skey* = *[skey1, skey2, …, skey3]* to encrypt the corresponding data blocks, respectively. Different *skey* keys set different access control structures according to different permission requirements for data protection to meet the fine-grained access control requirements for data. Subsequently, the data encryption key *skey* is encryption protected based on the attribute encryption of the ciphertext strategy^19^.
2. Initialization of the public key and the master key: the bilinear mapping technology is applied to implement the multiplicative cyclic groups *G*_*0*_ and *G*_*1*_ selected by the authorized center, where *g* represents the generator of the cyclic groups *G*_*0*_ and *G*_*1*_, and *m* denotes the prime rank of groups *G*_*0*_ and *G*_*1*_. The bilinear mapping *e* is defined as *G*_*0*_ × *G*_*0*_* → G*_*1*_. The random numbers *R*_*α*_ and *R*_*β*_ are selected. *AAn* represents the *AA* node in the distributed environment, which generates the corresponding random parameter *rn ∈ Zm*. The public encryption parameter between *AA* is *PAA*, the authorization center generates the public key *PK,* and each *AA* generates the master key *MKn*^20^.
  1 $$ P_{AA} = e(g,g)\sum\nolimits_{n \in AA} {r_{n} } $$
  2 $$ PK{ = }\left\{ {G_{0} ,g,h = g^{Ra} ,e(g,g)^{{\sum {r_{n} } }} } \right\} $$
  3 $$MK_{n} { = }\left\{ {\beta r_{n} } \right\} $$
3. User key generation stage: the inputs of this stage include the key *PK* and the user attribute set *Auser*. The output is *SK*_*user*_. The random parameter *r* and the random parameter *ri* of attribute *i* are generated. Before the authorization center generates the key for the user, it needs to verify the user’s identity. Only when the verification result is a legitimate user, the authorization center can generate the key *SK*_*user*_ for the user.
  4 $$ SK_{user} { = }\left\{ {D = g^{{(\sum {r_{n} + r} )/r_{\beta } }} } \right\} $$
4. Data encryption stage: the inputs of this stage include the key *PK*, the *skey* that needs encryption input by the data owner, and the strategy tree *TACP*; the *CT* is output. The specific encryption process is illustrated in Fig. 4.

### Model performance verification

The model is simulated to verify its actual effects. The experiment setup is as follows: a computer with Windows10-64 bit operating system, 16G memory, a quad-core 2.7 GHZ processor, Intel Core i7-CPU (Central Processing Unit), and VMware virtual machine Software. Multiple 64bit Ubuntu operating system virtual machines are installed in Vmware for experiments. The Weil bilinear mapping algorithm in the PBC (Pairing-Based Cryptography) library function is adopted for the experiment. The needed MPAL (Multiple Precision Arithmetic Library) and glib function library are installed in the Ubuntu virtual machines. Simulation experiments are based on the open-source CP-ABE (Ciphertext Policy Attribute-Based Encryption) software package. The same experiment is repeated 20 times, and the averaged result is taken as the final experiment record. Derwent Innovation, State Intellectual Property Office of the Republic of China, and Iprdaily are used to collect and analyze data on blockchain technology patents.

## Results and discussions

### Scheme security analysis

#### Data privacy security

**Figure 5:**
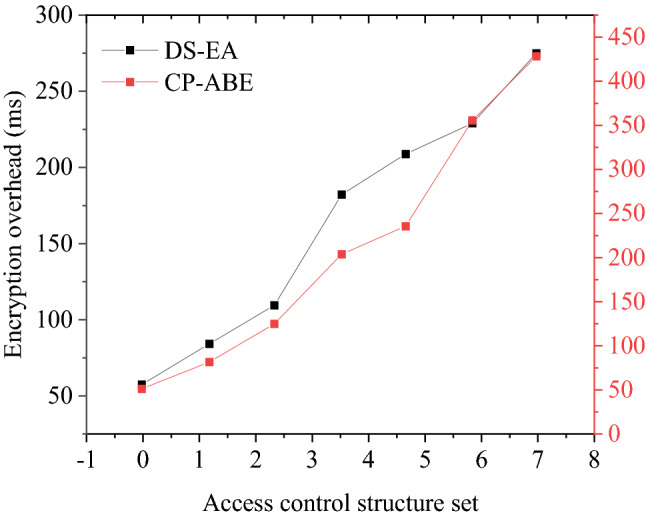
The relationship between the number of attributes and encryption overhead.

As shown in Fig. 5, different access control structures have a greater impact on the data encryption rate. Among them, the speed of data file encryption and decryption is associated with the access control structure. The more complex the access control structure, the slower the encryption and decryption rate. On the contrary, the simpler the structure, the faster the encryption and decryption rate. With the increasing number of attributes involved in the access control strategy, the time used for data encryption operations gradually increases. However, the increase is almost stable, indicating that the increase in overhead is acceptable. This result shows that the distributed storage of patent data is safe, and it can meet the requirements of off-site storage for the review.

#### Data operator security

**Figure 6:**
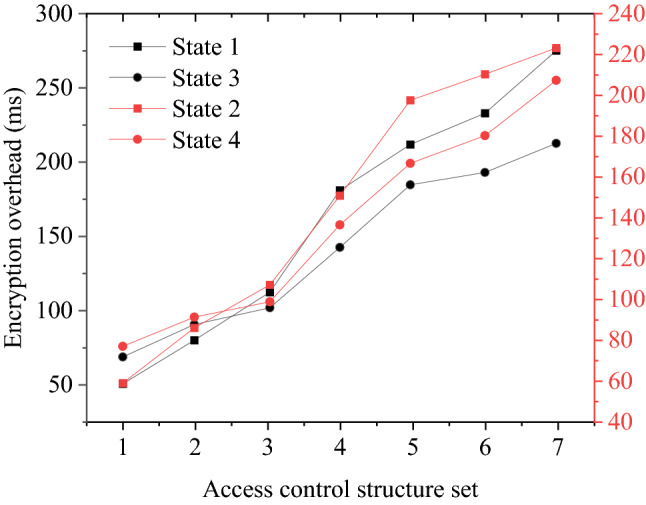
The relationship between the number of attribute authority and encryption overhead.

As shown in Fig. 6, different data operators correspond to the number of attribute authority data of 1–4. The difference in the number of attribute authorities has a significant impact on the calculation time-delay of the encryption parameter. The more the number of attribute authorities, the more time overhead the encryption needs; the more attribute authority, the more corresponding attribute management sets, and the more parameters need to be managed. This result also shows that the data operator cannot obtain patent data, steal data resources, or cause data leakage.

#### Data manager security

**Figure 7:**
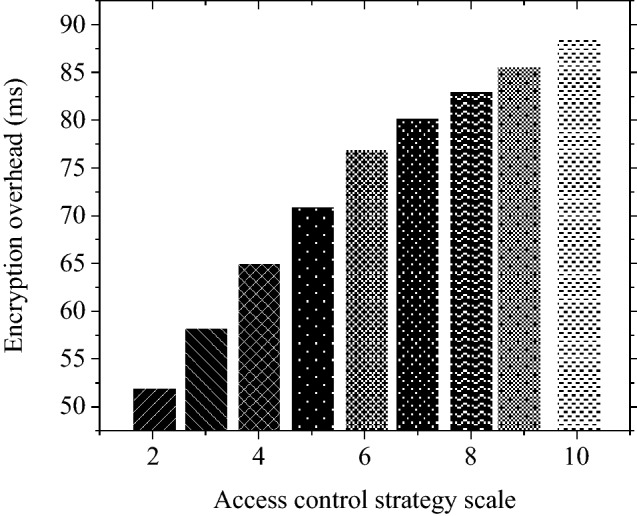
The relationship between the number of permissions and the encryption overhead.

As shown in Fig. 7, the number of permissions is associated with data encryption time-delay. The more permissions the data owner needs to manage, that is, the more access control strategies, the larger the encryption overhead. Therefore, even if there are more data managers, the specific information of patent data cannot be obtained effectively. Consequently, more managers are required to grant relevant permissions to access the data content.

#### Data owner security

**Figure 8:**
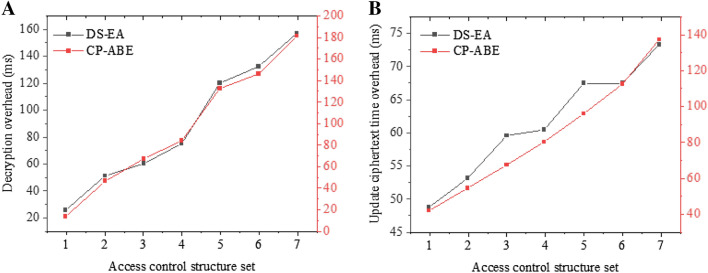
Overhead of ciphertext time after attribute decryption and update (**A** Decryption overhead; **B** Update ciphertext time overhead).

Figure 8A shows the time required to decrypt the file, and Fig. 8B shows the time required to update the ciphertext. The more attributes involved in the data decryption time-delay and ciphertext, the greater the overhead of decryption. Due to the distributed attribute management architecture and the ciphertext update calculation process, only part of the ciphertext needs to be updated when the attribute is updated, which effectively reduces the update time of the ciphertext after the attribute is updated. The ciphertext update time-delay and the classic CP-ABE encryption mechanism^21^ have been improved significantly. The data owners establish a representative of security services, effectively preventing data leakage from storage product vendors, data management vendors, and system vendors. The traceability and non-tampering characteristics of blockchain are used. Through the blockchain transaction management to access the control strategy and attributes, this function realizes the strategy management and tracking of the whole process of policy publishing, updating, and revocation. The strategy is stored in the blockchain in an open and transparent form. Any user can query it. The query function is separated from the traditional access control service mode by the third party. This function solves the problem of transparency of jurisdiction judgment.

### Model performance analysis

#### Computing overhead analysis

**Figure 9:**
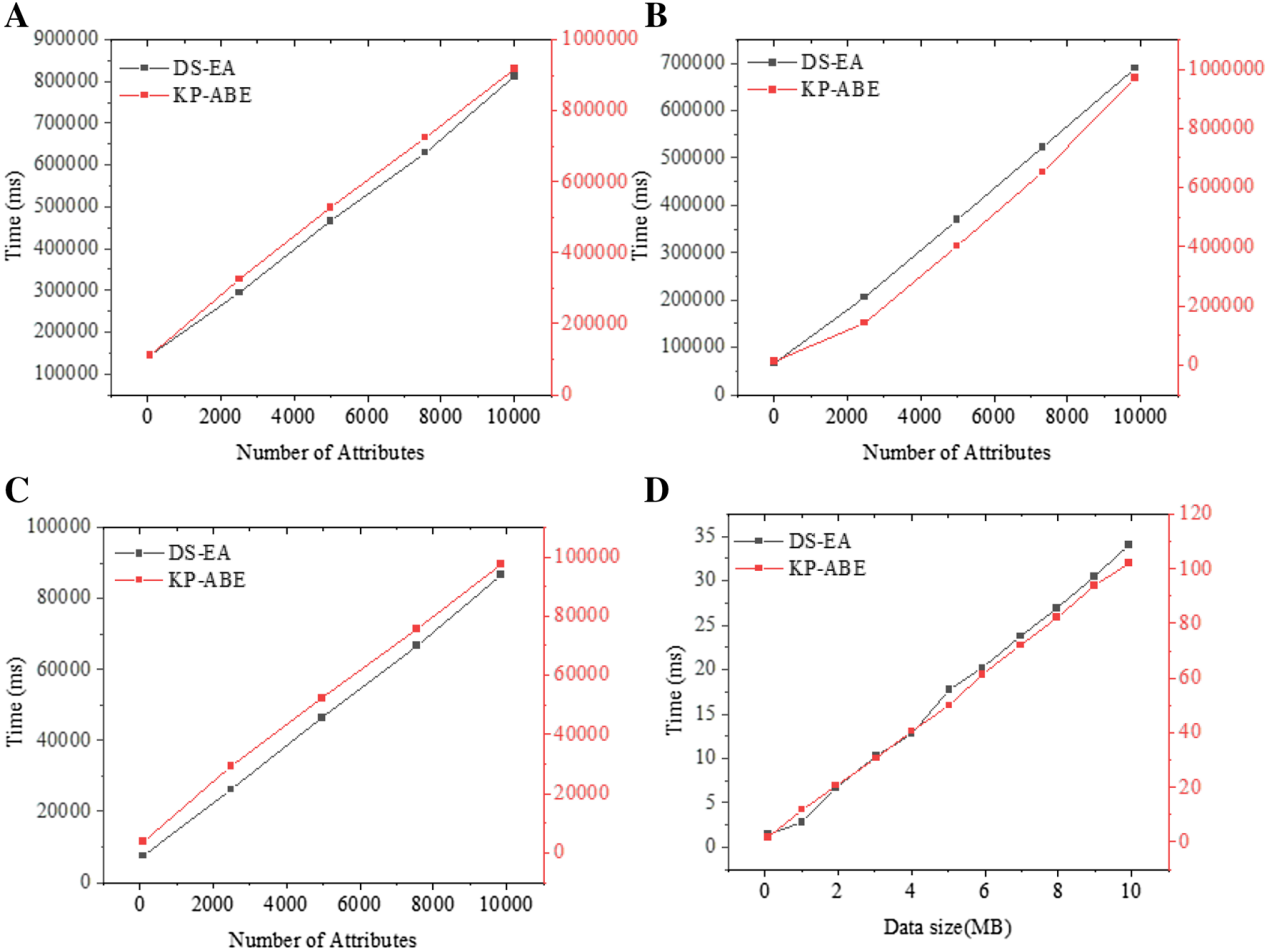
Computing overhead performance analysis (**A** Key overhead; **B** Encryption overhead; **C** Decryption overhead; **D** computing overhead).

Figure 9A–D demonstrate the key overhead, encryption overhead, decryption overhead, and computing overhead under different datasets. The proposed model is compared with the KP-ABE (Ciphertext Policy Attribute-Based Encryption) algorithm^22^. The overheads of the proposed model’s encryption algorithm and the KP-ABE algorithm all increase linearly with the increase in the number of attributes. In the proposed model, the overhead of the key generation algorithm increases linearly as the number of attributes increases. In the KP-ABE algorithm, the overhead of the key generation algorithm increases exponentially as the number of attributes increases. In the proposed model, the overhead of the decryption algorithm is lower than the overhead of the encryption algorithm. This is because the decryption algorithm takes less exponential operations. The time required to encrypt a 10 MB file with 64-bit data and 128-bit data is 35 ms and 105 ms, respectively. The results of all experiments show that using the local resources in branches for decryption can reduce the cloud computing overhead of the patent office.

#### Storage overhead analysis

**Figure 10:**
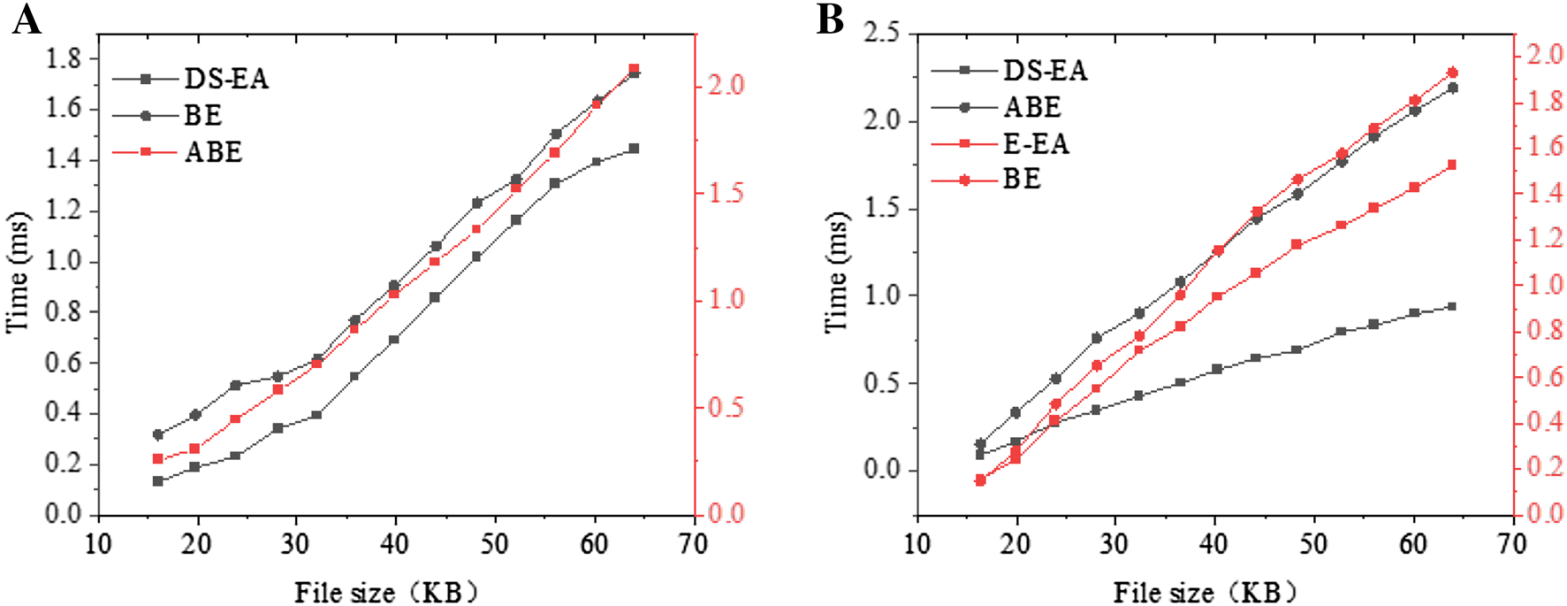
Storage overhead performance analysis (**A** The encryption algorithm; **B** The decryption algorithm).

Figure 10A displays the overhead of the encryption algorithm, and Fig. 10B displays the overhead of the decryption algorithm. DS-EA and BE-based schemes cost the least. Compared with the scheme based on ABE (Attribute-Based Encryption) and BE (Based Encryption) schemes, DS-EA can considerably reduce the key storage overhead. In this scheme, users only need to store their private keys and system parameters. In comparison, users must store their access structure and the corresponding private keys in the ABE-based scheme. Therefore, DS-EA only needs a small key storage overhead to implement secure cloud data collaboration services.

#### Network overhead analysis

**Figure 11:**
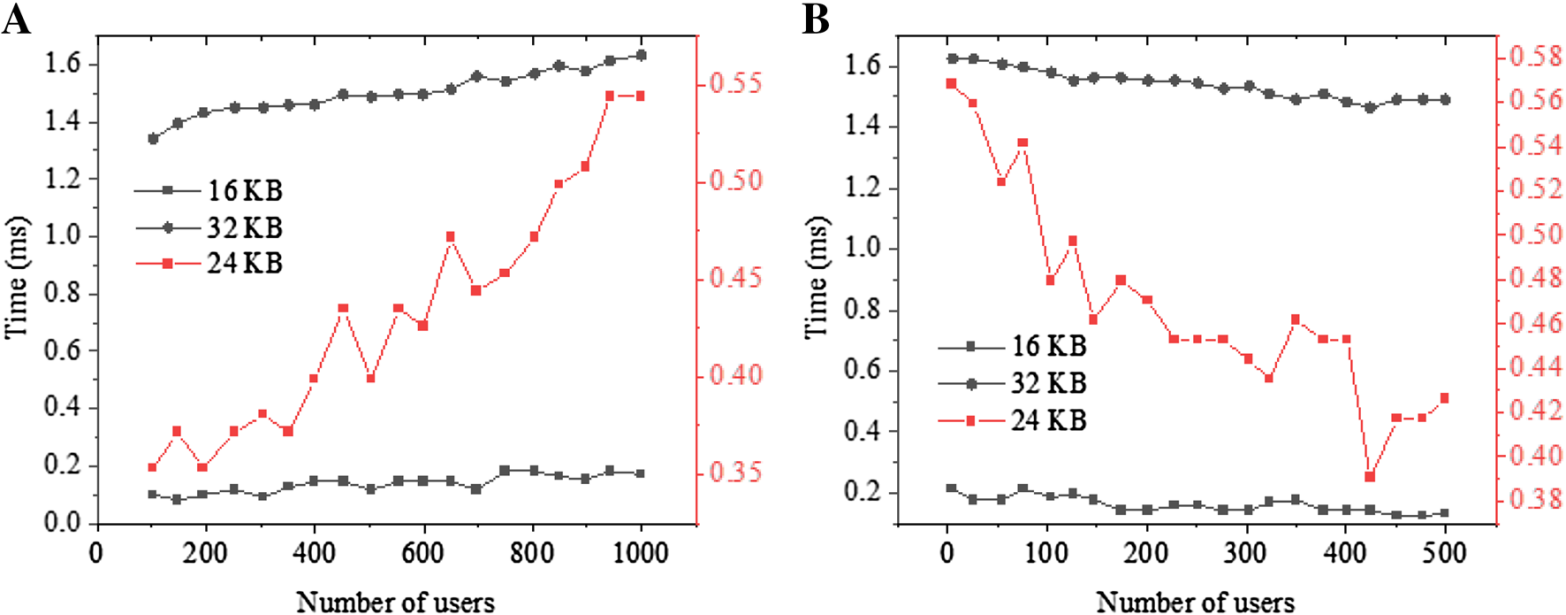
Time overhead of the encryption algorithms in SECO, ABE-based scheme, and BE-based scheme (**A** The encryption algorithm; **B** The re-encryption algorithm).

Figure 11A shows the network overhead of the encryption algorithm, and Fig. 11B shows the network overhead of the re-encryption algorithm. The proposed scheme only takes 1 s to decrypt the 64 KB data; in contrast, the algorithm proposed in previous research takes 1.5 s. Although the proposed scheme’s decryption algorithm must perform a pairing operation for each piece of data, the operation only needs to be done once, and the calculation can be completed at the very beginning. As the number of receivers increases, the encryption time-consumption is almost stable. Therefore, the DS-EA scheme is easy to expand in cloud computing. Experimental results show that DS-EA is lightweight and can apply to practice efficiently. This algorithm can reduce the storage space of the patent office encryption data and save the storage effectively.

#### Encryption performance analysis

**Figure 12:**
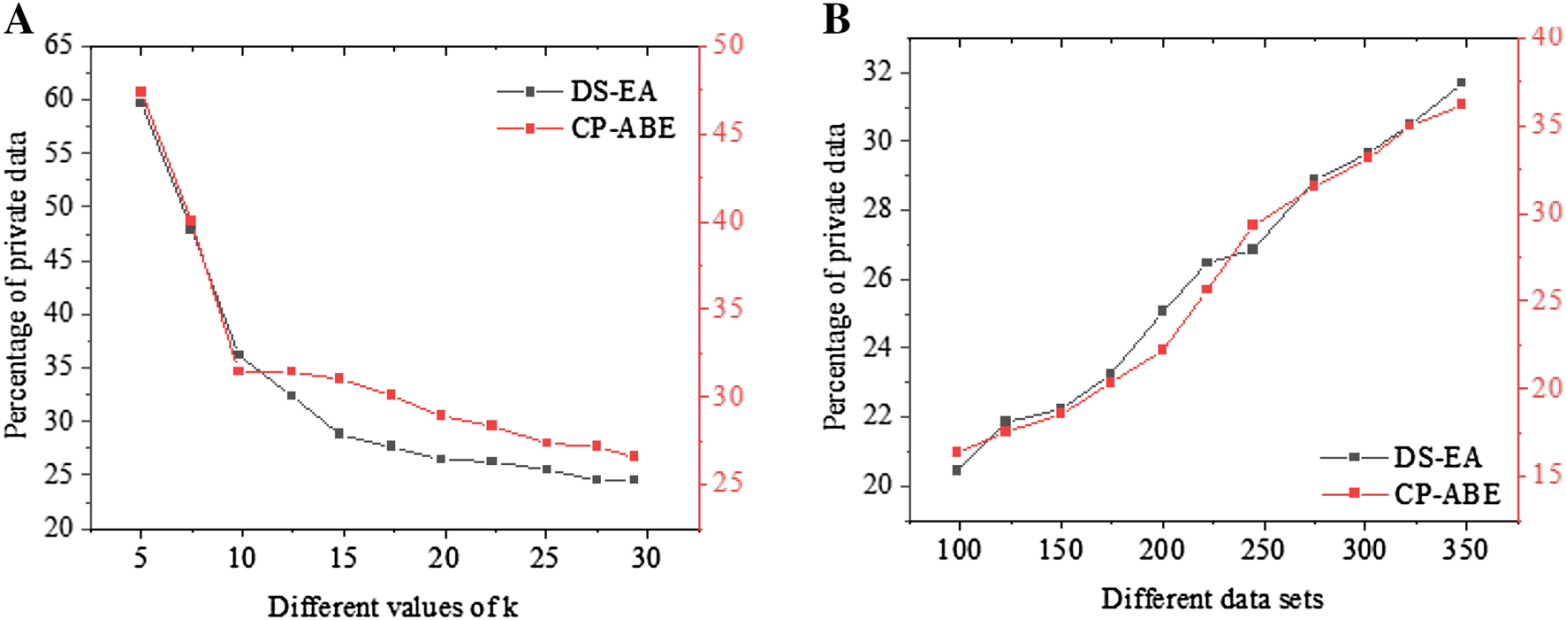
Percentage of users with privacy leaks under different k values and dataset sizes (**A** Under different k values; **B** Under different datasets).

Figure 12A illustrates the encryption performance results under different k values, and Fig. 12B presents the encryption performance results under different datasets. Only 1% of the data requires asymmetric encryption, which greatly reduces encryption computing overhead while increasing encryption speed and ensuring data security. Compared with the state of the art algorithms, the proposed algorithm has prominent advantages when the K value is large.

#### Test performance analysis

**Figure 13:**
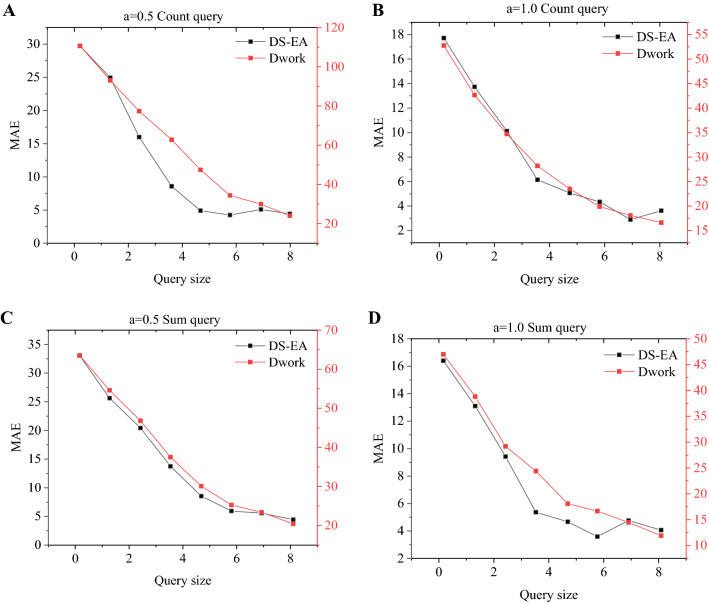
The MAE of different query sizes under different privacy (**A** a = 0.5 Count query; **B** a = 1.0 Count query; **C** a = 0.5 Sum query; **D** a = 1.0 Sum query).

**Figure 14:**
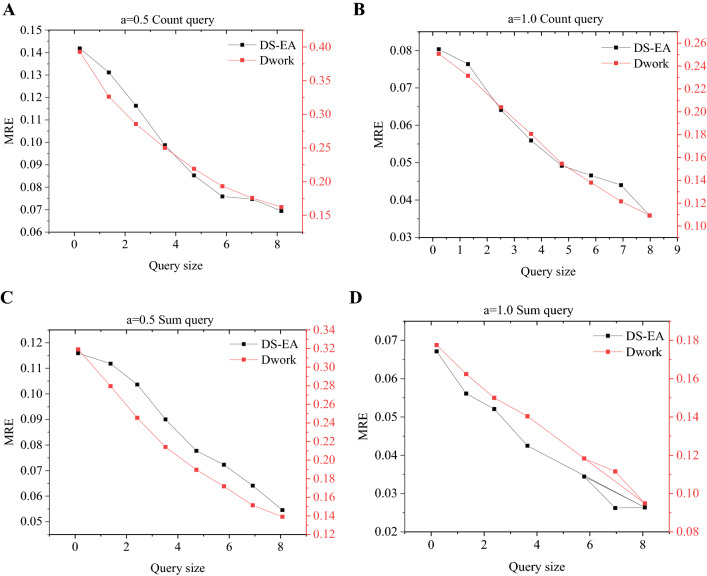
The MRE of different query sizes under different privacy (**A** a = 0.5 Count query; **B** a = 1.0 Count query; **C** a = 0.5 Sum query; **D** a = 1.0 Sum query).

Figure 13A–D represent the model’s MAE (Mean Absolute Error) results under *a* = 0.5 Count query, *a* = 1.0 Count query, *a* = 0.5 Sum query, and *a* = 1.0 Sum query. Figure 14A–D represent the model’s MRE (Mean Relative Error) results under *a* = 0.5 Count query, *a* = 1.0 Count query, *a* = 0.5 Sum query, and *a* = 1.0 Sum query. In any case, whether it is MAE or MRE, the results of the proposed algorithm are smaller than those of the Dwork algorithm^23^. When the query size is equal to 3 and *a* = 0.5, the MAE of the Count query result of the proposed algorithm is less than 20; in contrast, the result of the Dwork algorithm is close to 70. When the query size is 4 and *a* = 0.5, the MRE of the Sum query result of the proposed algorithm is less than 0.1; however, the result of the Dwork algorithm is greater than 0.2. As the query size increases, not only the MAE but also the MRE are decreasing. In addition, as *a* increases, both MAE and MRE are decreasing.

**Figure 15:**
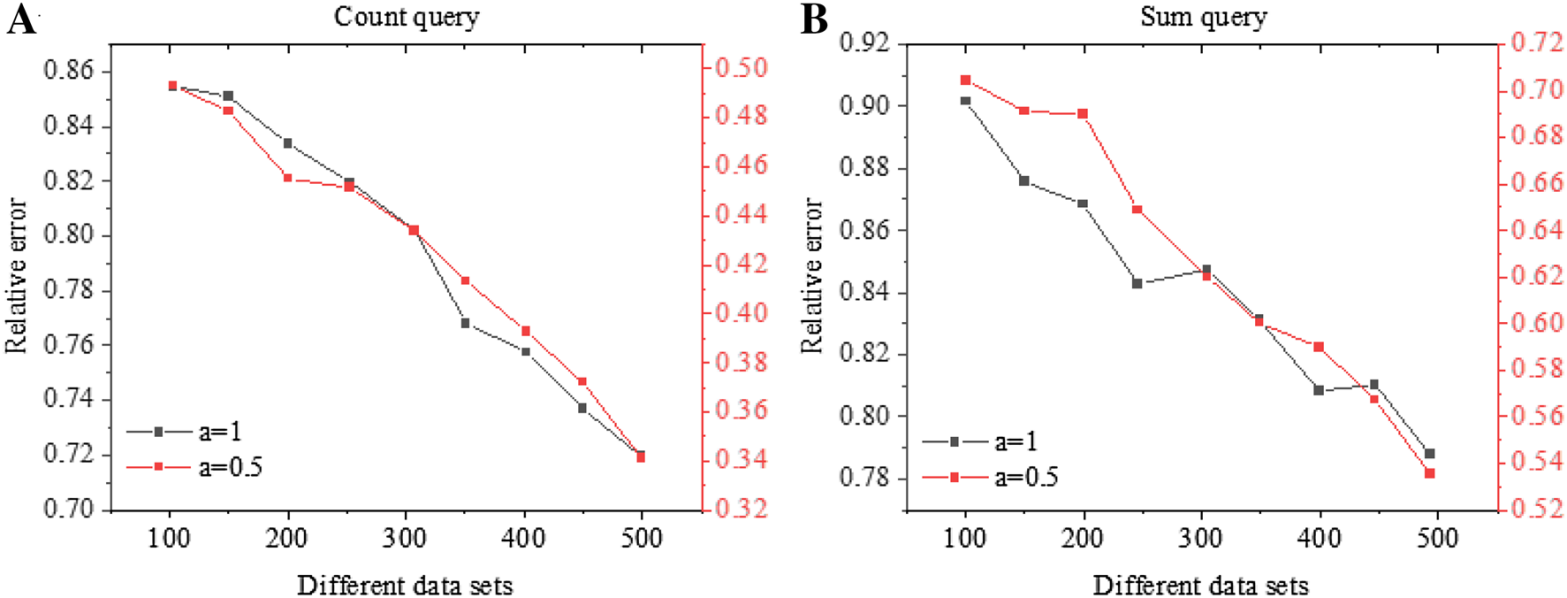
Relative error ratio under different privacy budgets and dataset sizes (**A** Count query; **B** Sum query).

Figure 15A presents the model’s relative error result under the Count query, and Fig. 15B gives the model’s relative error result under the Sum query. As the size of the dataset increases, the relative error ratio decreases. As the dataset grows to 1,500,000, and *a* = 0.5, the relative error ratio of the Sum query result is 0.7; when the dataset size is 4,500,000, the relative error ratio is less than 0.6. Therefore, the algorithm can provide higher data availability for large-scale multidimensional datasets.

## Conclusions

A scheme for patent data access control and protection is proposed based on blockchain technology under the blockchain scenario. The proposed scheme combines data fragmentation and data encryption technologies to protect patent data privacy and content effectively. The examples prove that the scheme is safe and reliable in terms of privacy protection. The performance analysis of the proposed model further proves that the proposed scheme is efficient and feasible in actual application scenarios. Although a useful patent data and access control model has been constructed, several shortcomings are found. Blockchain-based privacy protection can improve the efficiency of shared storage and private data processing, but the security performance will be affected. Therefore, how to balance the safety performance while improving efficiency is a further problem to be studied. In addition, in the designed composite model, the access control strategy needs to be further improved according to the needs of users, to further improve the efficiency of patent data protection.
